# Dosage compensation is less effective in birds than in mammals

**DOI:** 10.1186/jbiol53

**Published:** 2007-03-22

**Authors:** Yuichiro Itoh, Esther Melamed, Xia Yang, Kathy Kampf, Susanna Wang, Nadir Yehya, Atila Van Nas, Kirstin Replogle, Mark R Band, David F Clayton, Eric E Schadt, Aldons J Lusis, Arthur P Arnold

**Affiliations:** 1 Department of Physiological Science, University of California, Los Angeles, CA 90095, USA; 2 Department of Medicine, David Geffen School of Medicine, University of California, Los Angeles, CA 90095, USA; 3 Department of Cell and Developmental Biology, University of Illinois, Urbana, IL 61801, USA; 4 W.M. Keck Center for Comparative and Functional Genomics, University of Illinois, Urbana, IL 61801, USA; 5 Rosetta Inpharmatics, Seattle, WA 98034, USA

## Abstract

**Background:**

In animals with heteromorphic sex chromosomes, dosage compensation of sex-chromosome genes is thought to be critical for species survival. Diverse molecular mechanisms have evolved to effectively balance the expressed dose of X-linked genes between XX and XY animals, and to balance expression of X and autosomal genes. Dosage compensation is not understood in birds, in which females (ZW) and males (ZZ) differ in the number of Z chromosomes.

**Results:**

Using microarray analysis, we compared the male:female ratio of expression of sets of Z-linked and autosomal genes in two bird species, zebra finch and chicken, and in two mammalian species, mouse and human. Male:female ratios of expression were significantly higher for Z genes than for autosomal genes in several finch and chicken tissues. In contrast, in mouse and human the male:female ratio of expression of X-linked genes is quite similar to that of autosomal genes, indicating effective dosage compensation even in humans, in which a significant percentage of genes escape X-inactivation.

**Conclusion:**

Birds represent an unprecedented case in which genes on one sex chromosome are expressed on average at constitutively higher levels in one sex compared with the other. Sex-chromosome dosage compensation is surprisingly ineffective in birds, suggesting that some genomes can do without effective sex-specific sex-chromosome dosage compensation mechanisms.

## Background

In diploid animals with heteromorphic sex chromosomes (that is, where the sex chromosomes differ in gene content), males and females have a different genomic dose of sex-chromosome genes. In mammals, for example, which have X and Y sex chromosomes, there are two copies of the X genes in females (XX) compared with one copy in males (XY). The twofold difference in genomic dose of an entire chromosome is thought to present a serious potential problem. Because X and autosomal (A) genes interact within gene networks, a sexual imbalance of X and A gene doses would compromise development and function in at least one of the sexes [1]. Different animals have evolved different molecular mechanisms to balance X and A gene dose in the two sexes. Mammals inactivate one X chromosome in females and increase the expression of X genes in both sexes to be on par with that of the A genes [2,3], *Drosophila *increases transcription from the single X in males [2,4], and *Caenorhabditis elegans *reduces transcription of genes on both X chromosomes in females, but achieves X versus A parity by increasing X expression in both sexes [1,4]. The convergent evolution of different molecular mechanisms to achieve dosage compensation suggests that effective dosage compensation is critical, and perhaps ubiquitous, among species with heteromorphic sex chromosomes [3-5]. Dosage compensation involves two processes: sex-specific dosage compensation (SSDC), a chromosome-specific (and often chromosome-wide) mechanism of equating X dosage in the two sexes; and a mechanism to adjust X dose to A dose in both sexes [3]. It is not yet clear whether these two processes, which are conceptually distinct, necessarily involve different molecular mechanisms [5].

Dosage compensation has received comparatively little attention in birds, in which the male is homogametic (ZZ) and the female heterogametic (ZW). Three previous studies measured the male to female (M:F) ratio of 11 Z genes, mostly in chickens (*Gallus gallus*) [6-8], and found that Z genes had M:F expression ratios ranging from 0.8 to 2.4. If one assumes that compensated genes would show a M:F ratio near 1, and that non-compensated genes would have a ratio near 2, these studies suggest that some Z genes are dosage compensated but others are not. Other studies have found disproportionate numbers of Z genes with higher expression in males than in females, fueling speculation that the Z genes may not be completely dosage compensated [9-13].

For several reasons these studies do not resolve whether, or how much, dosage compensation occurs in birds. First, studies of aneuploid systems in maize and *Drosophila *indicate that differences in copy number of parts of a chromosome do not lead to a proportional change in gene expression [14]. For example, when different *Drosophila *strains with 1.5- or 3-fold differences in the copy number for a segment of an autosome were compared, genes encoded by that segment were expressed on average about 1.2- and 1.5-fold differently, respectively [4]. In the absence of evolved mechanisms for chromosome-wide dosage compensation, the fold-change in expressed gene dose is often considerably less than the difference in gene copy number in the genome [14,15]. Thus, even if there were no SSDC of the avian Z chromosome, one would expect that M:F ratios of Z expression would be less than 2. Even when chromosome-wide SSDC such as X-inactivation occurs, some genes escape inactivation and are more highly expressed in the homogametic sex [16,17], so the finding of a few Z genes in birds with greater expression in males than females does not mean that Z-chromosome SSDC does not occur. Moreover, previous studies did not measure sufficient numbers of genes to give an impression of typical M:F ratios for Z genes, and did not compare the M:F ratios of Z and A genes to determine whether they differ in their sex ratios and if Z:A parity is achieved. Here we examine these questions using larger sets of genes in two bird species, zebra finch (*Taeniopygia guttata*) and chicken, and compare the results with similar analyses for mice and humans.

Complete inactivation of one of the chicken Z chromosomes seems unlikely, because both alleles of Z genes are expressed in males, according to two studies that measured a total of seven genes [8,18]. Differential expression of Z genes in the two sexes could be controlled by a non-coding RNA that is transcribed from the female, but not the male, chicken Z chromosome, in a region that is hypermethylated in males but not in females [19,20]. The non-coding RNA is not translated but accumulates as a high molecular mass RNA at the site of its transcription on the Z chromosome of females. Non-translated RNAs are also involved in SSDC in other species, such as the *Xist* RNA in mammals and *roX1* and *roX2* RNAs in *Drosophila*.

Previous analyses confirm theoretical expectations that the sex chromosomes contain specialized functional sets of genes [21-25]. In XX-XY systems, the male-specific Y genes spend all of their evolutionary history in males, and have evolved male-specific functions. X-chromosome genes are subject to competing evolutionary pressures. Although X genes good for males are immediately subject to positive selection because of their hemizygous exposure in males, X genes also spend twice as much of their evolutionary history in females as in males, so that they may be under differential selection to be good for females. The X chromosome of *Drosophila *has relatively few genes that are involved in male reproduction (supporting the idea of feminization of the X), whereas in mammals the X chromosome has accumulated genes involved in brain, muscle, and reproductive functions, as well as genes acquired by retrotransposition [23]. Because specialization of the gene content of the Z chromosome appears to occur in birds [26,27] and could shift M:F ratios of gene expression [22], we also sought evidence for specialization of the Z chromosome.

## Results and discussion

### Analysis of male:female ratios of gene expression in the zebra finch

**Figure 1:**
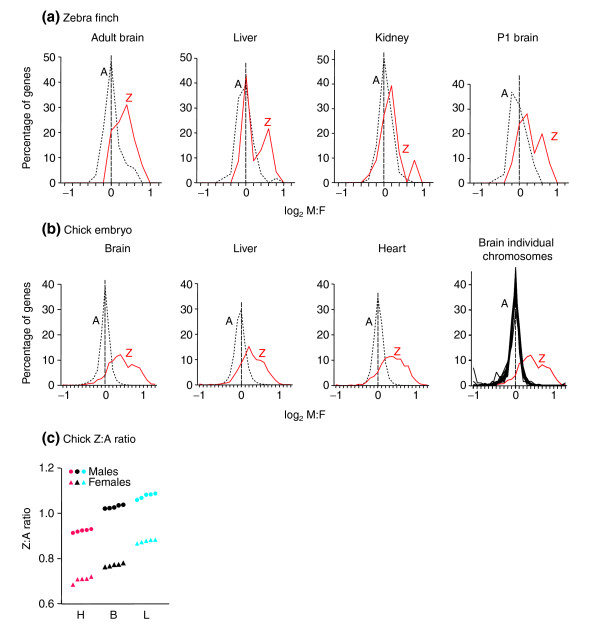
Distributions of male-to-female (M:F) ratios of gene expression based on microarray studies of birds. **(a) **M:F ratios in zebra finches, in adult brain, liver, and kidney, and brain of post-hatch day 1 (P1). Autosomal genes (A) are represented by the black dotted line, Z genes (Z) by the red line. The vertical dashed line is centered at a M:F ratio of 1 (log_2 _ratio of 0). **(b) **M:F ratios of embryonic chick brain, liver and heart. In each case Z genes are expressed at higher M:F ratios than A genes. In (b) the panel on the far right shows distributions for brain of individual chromosomes containing more than 50 genes. In all panels in (a) and (b) the rightmost bin (at the rightmost mark on the abscissa) includes all genes with M:F ratios at that value or greater, and the leftmost bin includes all genes with M:F ratios at that value or smaller. **(c) **Z:A ratios of five male and five female chicken samples for heart (H), brain (B) and liver (L).

We first constructed a small cDNA microarray for the zebra finch with probes for A and Z genes, but enriched in Z genes. Of 131 expressed sequence tags (ESTs) spotted onto the arrays and used in the present analysis, 84 were classified as A and 40 as Z (see Materials and methods). M:F ratios of expression were calculated from hybridization of male versus female samples. In each of four tissues (adult brain, kidney, liver, and post-hatch day 1 (P1) brain), the log_2 _M:F ratio was significantly greater in Z genes compared with A genes (Mann-Whitney U test: *p *< 0.00002 for adult and P1 brain, *p *< 0.0002 for liver, *p *< 0.02 for kidney; Figure 1a and Table 1). Moreover, the distributions of M:F ratios for Z versus A genes were significantly different (Kolmogorov Smirnov (KS) two-sample test, *p *< 0.001 for adult and P1 brain, *p *< 0.006 for liver, *p *< 0.05 for kidney).

**Table 1:** Male:female ratios of expression for A and X or Z genes in four species

Species and tissue	A mean	A median	X or Z mean	X or Z median	Male X:A or Z:A ratio	Female X:A or Z:A ratio
Mouse						
Brain	1.00	1.00	0.997	0.997	1.30	1.28
Muscle	1.01	0.990	0.967	0.960	0.866	0.875
Liver	1.03	0.993	1.01	0.975	0.726	0.729
Adipose	1.02	0.976	1.04	0.989	0.768	0.755
						
Human						
Hypothalamus	1.03	1.01	1.01	1.000	1.03	1.05
Muscle	1.02	1.01	0.990	0.989	1.14	1.26
LB cells	1.00	1.00	0.973	0.987	N/A	N/A
PBM cells	1.00	1.00	1.00	1.001	0.979	0.981
						
Zebra finch						
Adult brain	1.04	0.988	1.29	1.25	N/A	N/A
P1 brain	0.994	0.957	1.23	1.16	N/A	N/A
Liver	0.984	0.950	1.19	1.07	N/A	N/A
Kidney	1.04	1.01	1.13	1.09	N/A	N/A
						
Chicken						
Brain	0.997	1	1.40	1.34	1.03	0.771
Heart	1.02	1.02	1.34	1.31	0.923	0.707
Liver	0.989	0.978	1.24	1.20	1.08	0.877

The table shows unlogged values.

**Figure 2:**
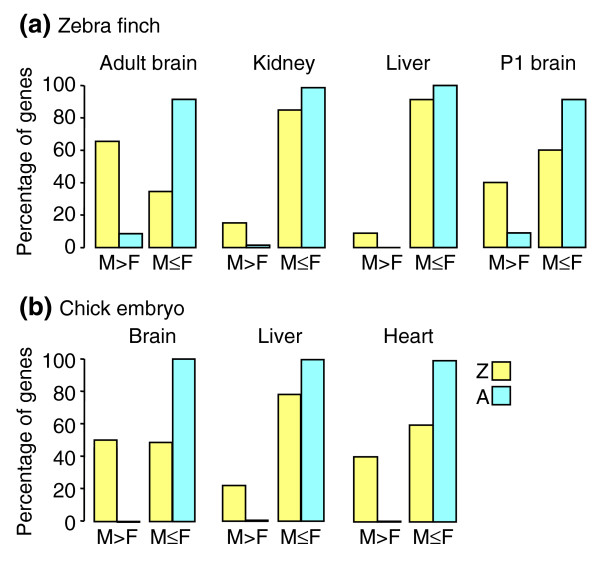
Comparison of male and female gene expression in birds. The bar graphs compare the percentages of Z (yellow) and A (blue) genes that are expressed at significantly higher levels in males vs females (M > F) or are expressed equally or more highly in females (M ≤ F). Four tissues are shown for zebra finch **(a) **and three for chick embryo **(b)**. In all tissues, a significantly greater proportion of Z-linked genes, relative to A genes, were expressed at higher levels in males than females.

We performed a gene-by-gene analysis to determine which genes showed a significant sex difference in expression. Of 52 cases in which expression of a gene in individual tissues was found to be sexually dimorphic and significant at a 10% false discovery rate (FDR), four genes (all A) were expressed more highly in females. Of the other 48 genes, expressed at a higher level in males, 36 were Z genes (Figure 2). Genes expressed at a significantly higher level in males were disproportionately found to be Z-linked in all tissues except liver (*p *< 0.00000001 for adult brain, *p *< 0.02 for kidney and P1 brain, *p *= 0.08 for liver, Fisher's Exact Test). Twenty-one genes (16 Z, 5 A) were found to be expressed at a significantly higher level in males in two or more tissues (Table 2), a result that increases the likelihood that these genes were not false positives. The M:F expression ratio of Z genes was correlated across the four tissues (mean pairwise *r *= 0.75, range 0.59–0.86), suggesting that the M:F ratio was influenced by regulatory factors that operate in multiple tissues.

**Table 2:** Zebra finch genes showing sex difference in more than one tissue

GenBank accession number	Gene symbol	Category	Brain	Kidney	Liver	P1 brain
CK311497	*CBWD1*	Z	3.6E-03	5.3E-02		7.8E-02
	*Cyclophilin*	A	3.8E-02			5.5E-02
CK315180	*DNAJA1*	Z	2.3E-04	2.0E-03	3.1E-04	6.6E-03
DV959526	*ERCC8*	Z	2.8E-02			4.9E-02
CK304072	*FST*	Z	4.4E-05		5.2E-02	1.2E-02
CK313884	*HSD17B4*	Z	7.5E-06	2.6E-05	2.7E-02	3.2E-03
CK304237	*LUZP1*	Z	5.1E-03			1.1E-02
CK310795	*MCCC2*	A	2.1E-02	1.5E-04		
CK311614	*PSIP1*	Z	5.7E-02			9.8E-02
CK310447	*RIOK2*	A	5.8E-02			3.5E-03
CK316148	*RPS6*	Z	2.9E-05	5.6E-07	8.5E-03	2.0E-04
DV958433	*SMARCA2*	Z	5.6E-04	2.6E-03		6.3E-02
DV946673	*TARS*	Z	1.4E-02		9.3E-02	
DV948056	*TNPO1*	Z	2.4E-02			2.1E-02
CK314627	*UBAP2L*	A	4.5E-04			1.3E-02
DV946458	*UHRF2*	Z	1.0E-02		8.1E-02	
DV955869		Z	2.1E-06	2.2E-02		
CK313394		Z	3.0E-03	6.5E-04	2.6E-02	2.1E-03
DV956828		Z	6.6E-03			2.9E-02
CK305660		A	1.1E-04	2.0E-02		6.7E-04
DV953757		Z	6.9E-03			1.8E-03

The *P*-value for each tissue reflects the results of the paired *t*-test.

To confirm the result of the zebra finch microarray analysis, we used quantitative reverse transcription-PCR (RT-PCR) to measure the sex differences in expression of six Z-linked genes in adult and P1 brain (Table 3). All but one of the M:F ratios measured using RT-PCR were higher than those estimated using the microarray analysis, and in adult brain all ratios were close to 2. The lower ratios in the microarray analyses may be due to nonlinearity over the dynamic range of the signal intensities, or to other factors [28].

**Table 3:** Comparison of the analysis of gene expression by quantitative RT-PCR and microarray

		M:F (microarray)		M:F (RT-PCR)	
			
Species and tissue	Affymetrix number or gene symbol	Ratio	*P*-value	Ratio	*P*-value
Zebra finch					
Adult brain	*FST*	1.7	4.0E-04	1.8	5.0E-04
	*LUZP1*	1.6	3.7E-03	2.4	1.0E-06
	*SMARCA2*	1.4	1.7E-03	2.1	2.0E-06
	*DNAJA1*	1.3	1.6E-03	2.0	4.6E-05
	*CRHBP*	1.2	5.9E-02	1.4	2.8E-03
	*RPS6*	1.6	2.4E-05	2.1	1.3E-04
					
P1 brain	*FST*	1.6	1.5E-02	1.5	3.0E-02
	*LUZP1*	1.7	1.8E-02	2.5	4.4E-04
	*SMARCA2*	1.2	1.7E-01	1.8	9.0E-04
	*DNAJA1*	1.1	2.1E-02	2.1	1.3E-05
	*CRHBP*	ND	ND	2.1	8.7E-04
	*RPS6*	1.6	2.4E-04	2.5	3.0E-06
					
Chicken					
E14 brain	Gga.12454.1.S1	0.4	4.9E-05	0.5	7.3E-05
	GgaAffx.9524	1.0	0.59	1.1	0.64
	GgaAffx.24493	1.6	5.0E-06	1.8	1.3E-03
	Gga.4811	1.8	9.9E-08	2.3	2.5E-04
	GgaAffx.25289	2.0	1.7E-06	2.7	9.0E-05
	Gga.2433	2.4	1.1E-07	2.6	2.9E-04
	Gga.2883	2.7	1.3E-08	2.6	1.2E-05

ND, not detected. The *P*-value is from a t-test comparing males and females.

### Global analysis of chick embryo gene expression

To determine whether the Z versus A difference in M:F ratios in the zebra finch is generalizable, we performed a more global analysis of gene expression in a second bird species, the chicken, using Affymetrix Chicken Genome microarrays, which measure the expression of more than 28,000 genes. Expression was analyzed in the brain, liver, and heart of chick embryos at day 14 (*n *= 5 biologically independent samples of each sex, each sample composed of RNA from three or four different birds). In the filtered dataset, M:F ratios were calculated for a total of 16,506 probes (827 Z, 15,679 A) in the liver, 17,757 (918 Z, 16,839 A) in the heart, and 18,920 (964 Z, 17,956 A) in the brain. The log_2 _M:F ratios for Z genes were clearly higher than for A genes in each tissue (Figure 1b, Table 1, and Additional data file 1), with *p *< 9E-219 in each case (Mann-Whitney U test) and the distributions of M:F ratios of A and Z genes differed in each tissue (*p *= 0, KS tests). The distributions of M:F ratios of individual autosomes were similar to each other but different from that for the Z chromosome (Figure 1b, right panel). The mean log_2 _M:F ratio was near 0 for each autosome (range for autosomes in brain -0.016 to 0.002, liver -0.047 to 0.133, heart -0.047 to 0.048, considering autosomes with > 50 probes). We used quantitative RT-PCR to establish that M:F ratios measured in the microarrays were accurate (Table 3). The results for chick tissues agree well with those for zebra finch.

Of 1,334 probes found to be sexually dimorphic in individual chick tissues, 1,180 (1,100 Z, 80 A) were higher in males and 154 (13 Z, 141 A) were higher in females. The proportion of genes expressed more strongly in males compared with females (M > F) was higher among Z genes than among A genes (*p *< 10E-15 for each tissue, Fisher's Exact Test, Figure 2). The M:F expression ratio of Z genes was correlated across the three tissues (pairwise *r *= 0.65 for brain/liver, *r *= 0.73 for brain/heart and heart/liver), suggesting that the M:F ratio was influenced by regulatory factors that operate in multiple tissues.

The M:F ratios for Z genes appear to have a bimodal distribution in several (but not all) tissues in zebra finch and chicken. Bimodality could be evidence for the existence of two discrete populations of Z genes that are more or less dosage compensated. However, the data do not show strong bimodality, because all of the distributions for zebra finches and chick embryo tissues were not significantly bimodal as assessed by the dip test [29].

As expected from the M:F ratios described above, the mean Z:A ratios for chicken brain, liver, and heart were consistently higher in males than females for each tissue, with no overlap in the values (Figure 1c). The Z:A ratio was higher in males than in females (33% higher in brain, 23% higher in liver, and 31% higher in heart, Table 1). The Z:A ratios are within the range of X:A ratios reported for mammals, suggesting that mechanisms have evolved to balance Z and A gene in expression in birds, as in mammals, although the balance is less effective in females than in males [3] (Table 1). Unlike the situation for X:A ratios in mammals, the Z:A ratio for brain is not higher than in other tissues [3]. We also analyzed previously published microarray expression studies utilizing a total of 52 arrays in five studies on chicken spleen/bursa, a macrophage cell line, embryonic and post-hatch pituitary, and peripheral blood macrophages. These analyses, on samples of unknown or mixed sex, show mean Z:A ratios of 0.780, 0.807, 0.987, 1.05, and 1.16, in the same range as our results for embryonic tissues.

To determine whether these data show specialization of gene content on the Z chromosome in chickens, we asked whether specific types of genes are concentrated on the Z chromosome compared with autosomes. When 'liver genes' were defined as those found in the filtered dataset for liver but not in the datasets for brain or heart, fewer liver genes were found on the Z chromosome than expected by chance. Of 765 genes classed as 'liver' according to this definition, 24 were on the Z, less than the 38 expected from the number of non-liver genes on the Z (803) relative to all non-liver genes (14,938) (Fisher's Exact Test, *p *= 0.014). In contrast, when liver genes were defined as genes found in all tissues but twofold higher in liver than in either of the other tissues, liver genes were found more often on the Z chromosome than expected by chance. In this case, of 156 liver genes, 17 were Z, more than the seven expected on the basis of the number of non-liver genes on the Z (204) relative to all non-liver genes (4,777) (*p *= 0.0003). Brain and heart genes, defined according to either of these methods or several others, showed no specific over- or under-representation on the Z chromosome. These results indicate that although one can find a definition that shows enrichment of liver genes on the Z chromosome relative to autosomes, not all definitions show that effect.

If concentration of male-biased genes on the Z chromosome, rather than the difference in genomic dose of Z genes, is responsible for the significantly higher M:F ratio of Z genes relative to A genes, one would predict that housekeeping genes would not show the Z versus A difference in M:F ratios. Housekeeping genes are important for function of both male and female cells, and therefore should not contribute to any Z versus A difference in M:F ratios caused by concentration of male-biased genes on the Z chromosome. To test this prediction, we selected for analysis housekeeping ribosomal and/or mitochondrial genes, those that contained the term "ribosomal" and/or "mitochondrial" in annotation of the probes on the Affymetrix chicken microarray. The set of ribosomal/mitochondrial genes comprised 224 genes (12 Z) in chick embryonic brain, 224 genes (11 Z) in heart, and 220 genes (12 Z) in liver. The mean M:F ratios were 1.58 Z:1.00 A for brain, 1.39 Z:1.00 A for heart, and 1.33 Z:0.981 A for liver. The results contrast with those for *Drosophila*, in which X and A ribosomal genes are expressed at about the same M:F ratios in gonads [30]. These results support the idea that the higher expression of Z genes in male birds is not simply a reflection of bias in the composition of the Z chromosome, but reflects ineffective dosage compensation.

### Comparison to mouse and human

**Figure 3:**
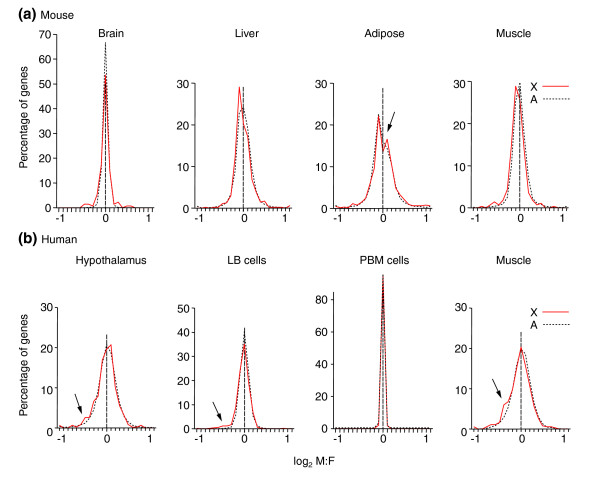
Comparison of male and female gene expression in mammals. In mouse **(a) **and humans **(b)**, each tissue has a distinct distribution of M:F ratios, but in each case the distribution for X genes (red line) fits closely to the distribution for A genes (dotted black line). LB, lymphoblastoid cell lines. PBM cells, peripheral blood mononuclear cells. Arrows point to regions where the X and A curves diverge, or to the inflection point in the mouse adipose tissue curve.

To compare these results for birds directly with those for vertebrate species in which the dosage compensation mechanism is better understood, we reanalyzed mouse and human microarray expression data from previous studies [31-36] (Figure 3a,b). In sharp contrast to the results for birds, the M:F ratios for X and A genes were quite similar within each tissue, although small X versus A differences in the distributions were sometimes observed. Moreover, the variability of the M:F ratios was different across tissues, unlike the situation in birds.

In mouse brain (see Figure 3a), the curve of the distribution of X genes had 'shoulders', unlike the A curve, suggesting that X genes were more likely to be sexually dimorphic – a disproportionate number of X genes had M:F ratios that were above or below the mode of the distribution, as reported previously [31]. In mouse muscle and liver, the distribution of M:F ratios for X genes was shifted slightly to the left relative to that of A genes (X vs A distribution *p *< 0.005 for liver and *p *< 0.001 for muscle, *p *> 0.05 for brain and adipose, KS test). The X vs A median M:F ratios were also significantly different in liver (*p *= 0.017) and muscle (*p *= 0.000005) but not in other tissues (*p *> 0.05, Mann-Whitney U) (Table 1).

In adult mouse brain, the variability of M:F ratios was smaller than in the other three mouse tissues, especially adipose [31], suggesting that both A and X genes were more equivalently expressed in male and female brain than in other tissues. The curves for adipose tissue were different from those in other tissues in that they had a clear inflection point for both X and A genes at an M:F ratio of 1 (log_2 _ratio of 0).

In the human tissues, peripheral blood mononuclear cells showed an extremely narrow range of M:F ratios, whereas the other human tissues showed broader distributions. Small X versus A differences were found in human hypothalamus, lymphoblastoid cells, and muscle, where proportionally more X genes appear to have lower M:F ratios (see Figure 3b, arrows). The X versus A distributions of M:F ratios were significantly different for lymphoblastoid cell lines (*p *< 0.001) and for muscle (*p *< 0.01) but not for the other tissues (*p *> 0.05, KS tests). The median M:F ratios for X and A genes were close to 1 for all tissues (Table 1), but differed between X and A in lymphoblastoid cells (*p *= 0.000003, Mann-Whitney U) and muscle (*p *< 0.003) but not for the other two tissues.

The slightly greater proportion of human X genes that were expressed higher in females than in males (see Figure 3b, arrows) could be explained by the escape from X inactivation found in human cells [16]. To analyze this question further, we examined the M:F ratios of X genes previously found to show some degree of escape from inactivation ('escapees') versus genes showing no escape (see Table 3 in [16]). Each gene was categorized as having a M:F ratio above or below 1. X escapees were found to be more likely than non-escapees to have ratios below 1 in lymphoblastoid cell lines (Fisher's Exact Test, *p *= 0.037), whereas in muscle and peripheral blood mononuclear cells, there was no sign of such a tendency (*p *> 0.4; brain could not be analyzed). Our results show that escape from X inactivation could contribute to lower M:F ratios, at least in some human tissues, although the effect is small when it is present. Thus, escape from inactivation has little effect on M:F ratios of X-gene expression in these studies (see also [3]). Tissue-specific differences in X inactivation have been reported previously in mice [37].

### Implications of the present results

The present results show a striking difference in dosage compensation in birds and mammals. In mouse and human, X inactivation and other dosage-compensation mechanisms result in remarkable parity of expression of X genes and A genes in the two sexes in a variety of tissues, despite the sexual inequality of X-chromosome genomic dose, as previously reported [3,4]. In contrast, in two bird species, Z genes are expressed at a consistently higher level in males than in females in several different tissues, including adult, embryonic, and neonatal tissues. The percentage difference in M:F ratios of Z versus A genes is as high as 40% among chicken tissues (Table 1). A 40% difference in expression might be considered minor in the case of an individual gene, but not when the difference is the average for the entire chromosome and half of the Z genes have M:F ratios above 1.34 as in the present case for chicken brain. The Z versus A difference in birds suggests that Z dosage compensation is ineffective, a surprising conclusion because sex-chromosome dosage compensation is thought to be critical and ubiquitous [3,4]. On the other hand, the Z:A expression ratios found in several bird tissues are in the range 0.71 to 1.08, which is close to the range of X:A expression ratios in mammals (Table 1 and [3]), indicating that some sort of compensation occurs that balances Z and A gene expression to some degree.

The data reported here may not be sufficient, by themselves, to invoke the existence of a dosage-compensation mechanism that is specific to the avian Z chromosome. Many gene networks, involving genes on all chromosomes, appear to have regulatory elements that are sensitive to dosage (for example, negative feedback, autoregulation, competition for a limited regulatory factor), and which generally act to mitigate the effects of differences in the copy number of the genes they regulate [5,15]. Differences in the genomic dose of genes lead to differences in expression of those genes that are generally less than the difference in genomic dose [4,14,38-40]. Thus, even in the absence of an evolved mechanism of SSDC for the avian Z chromosome, we would expect a distribution of M:F ratios with a mean less than 2, as observed here. Indeed, the magnitude of Z-chromosome dosage compensation found here is as large as previous estimates of the magnitude of autosomal network dosage compensation [4,38,39]. Only a few studies have estimated the magnitude of autosomal dosage compensation in vertebrates, however, so it is not clear whether the amount of Z compensation found here is compatible with a complete absence of SSDC. Moreover, a non-coding RNA is expressed in a sex-specific fashion from the chicken Z chromosome and is associated with female-specific acetylation of Z histones [19,20], suggesting that there may be sex-specific regulation of Z-chromosome gene expression. If SSDC occurs, it would have to be selective to fit the present data. Thus, we expect that it would disproportionately influence those genes for which sex differences would be particularly damaging, for example regulatory genes such as transcription/chromatin factors and signal transduction genes with a strong influence on the expression of other genes. Although this idea might help explain how birds have adapted to a constitutive sex difference in the expression of Z genes, one can find counterexamples of Z-linked regulatory genes that appear to have high M:F expression ratios (Additional data files 1 and 2).

The specialization of Z-chromosome gene content could provide an alternative model (to the difference in genomic dose of Z genes) to explain the higher M:F ratios for Z genes in birds. For example, Z genes might be more often good for males than for females, because they spend more evolutionary time in males [22,23,25]. If Z genes are not good for females, their expression might be adaptively reduced in females, thereby increasing M:F ratios. Several considerations suggest that such specialization of Z-chromosome gene content does not account for the male bias in Z-gene expression observed here. First, housekeeping genes thought not to be sex-biased, such as ribosomal and mitochondrial genes, show higher M:F ratios if they are Z-linked than A-linked, suggesting that genomic dose, not sex-biased function, is responsible. Second, although previous studies have demonstrated enrichment or a deficit of specific types of genes on the X chromosomes of various species, the amount of enrichment can be relatively small (for example, only 1–2% X vs A difference in gene concentrations in *Drosophila *[30]) and would not be expected to produce a 40% shift in mean M:F ratios. Third, although we have attempted to use the present data to find evidence for enrichment of chick liver, brain, or heart genes on the Z chromosome, the results so far do not suggest any pattern of tissue enrichment that would explain the higher M:F ratios of Z-gene expression. Finally, our analysis of mammalian M:F ratios shows that the well documented [22,23] specialization of X-gene content in mammals (for example, enrichment of female-benefit genes) is not associated with any major reduction in M:F ratios of X genes relative to A genes (Figure 3). Despite statistical enrichment of the X chromosome for brain and muscle genes in mammals, those genes probably do not dominate the population of X genes, so it remains an eclectic mix of genes. Thus, the mammalian data do not support the prediction that gene specialization will dramatically shift M:F ratios of avian Z genes relative to A genes. The large rightward shift of M:F ratios of Z genes relative to A genes in birds (Figure 1) is most likely to be the result of the sexual discrepancy in the genomic dose of Z genes.

Assuming that the Z chromosome contains genes that regulate the expression of autosomal genes in *trans*, then the (on average) 24–40% higher expression of Z genes in chicken male tissues (Table 1) would be expected to shift the expression of autosomal genes. If most regulatory genes inhibit rather than increase expression of other genes [14], autosomal genes might be shifted toward higher expression in females. There is little evidence for such a shift in the present data, because in chick embryonic tissues expression of autosomal genes is distributed approximately symmetrically around a mean M:F ratio of 1 (Figure 1b), and this symmetry is as good as, or better than, that for some of the autosomal distributions for mammalian tissues measured here in which no female shift is predicted (Figure 3). The lack of shift supports the idea that regulatory genes on the Z chromosome might be compensated more than those that have little effect on expression of other genes (that is, they have M:F ratios near 1), or that the regulatory genes have balanced positive and negative influences on autosomal gene expression.

The generally higher expression of Z genes in male versus female birds suggests that Z genes might be more likely to evolve a role in controlling sexual differentiation in birds, as compared with X genes in mammals. In order for a tissue to function differently in males and females, the expression of genes in the tissue must evolve sensitivity to one or more sex-specific factors [41]. One set of sex-specific factors is the gonadal hormones, which are widely available to tissues as signals that evolution can use to control sex differences. If diverse cells in the body of birds express many Z genes at higher levels in males than in females, then Z genes are also widely available as a set of sex-biased signals. Z genes have been proposed to regulate sexually dimorphic development of the zebra finch brain [13]. Some of the Z genes that are expressed at higher levels in zebra finch males than females, such as *FST*, *SMARCA2*, *LUZP1*, and *CRHBP*, are implicated in signal transduction or as regulators of transcription, and therefore are candidates for factors that induce sex differences in tissue function.

The similarity of the human and mouse M:F distributions is not entirely expected because more X genes are thought to escape X inactivation in humans than in mice. About 15–25% of all human X genes escape X inactivation at least partially, which could lead to higher X:A expression ratios in females [16]. The degree of escape from X inactivation has not been comprehensively studied in the mouse, but is thought to be lower than in humans, in part because XO mice are more viable and reproductive than XO humans [42,43]. Thus, we might have expected greater disparity of M:F ratios in X versus A genes in the human than in the mouse. The current results do not support such a difference (see also [3]). Although we found evidence for slightly lower M:F ratios of X genes reported to escape inactivation in human lymphoblastoid cells, there was little evidence for this in the other human tissues examined. Thus, the dosage compensation for these genes appears to be accomplished by a process other than X inactivation.

Birds clearly differ from mammals, *Drosophila*, and *C. elegans *in that less sexual parity of sex-chromosome gene expression is achieved. The current results are compatible with the existence of a sex-specific mechanism to equalize Z expression in the two sexes, for example by increasing Z expression in females or decreasing it in males. If such a sex-specific Z dosage compensation mechanism exists, however, it is selective for some Z genes (not chromosome-wide) and/or is relatively inefficient compared with that in mammals, *Drosophila*, and *C. elegans*.

Because Z:A ratios in birds are fairly close to 1, we conclude that some process must adjust Z and A gene expression to comparable levels. A mechanism of sex-specific dosage compensation of Z gene expression, as discussed above, could be responsible. We ask, however, whether it might also be possible that dosage-sensitive network regulatory compensation mechanisms, which operate in both sexes and are not targeted specifically to the Z chromosome [5,15], might be sufficient by themselves to explain the amount of compensation observed here.

## Materials and methods

### Zebra finch microarray

Because the chicken and zebra finch genomes have similar chromosome structure and roughly similar linkage of genes to specific chromosomes [44], we identified lists of candidate zebra finch Z and A genes based on chicken linkage, as reported in the summer 2004 draft sequence and annotation of the chicken genome (Gallus_gallus 1.0 [45]). Zebra finch ESTs homologous to the chicken Z and A genes were identified by BLASTing to the zebra finch brain EST database ESTIMA [46]. To determine whether the selected ESTs were Z- or A-linked, we hybridized ESTIMA cDNAs representing 131 genes to Southern blots of restriction-digested male and female genomic DNA. ESTs showing approximately equal hybridization in males and females were classified as A. ESTs with bands of approximately double intensity in males were classified as Z. Equal loading of lanes was checked by probing the blots with an autosomal gene, or by equal ethidium bromide staining of DNA in gels. We adopted a conservative criterion, so that only ESTs clearly showing greater representation in the male genome were classified as Z. Any incorrect assignment of A or Z genes would tend to decrease the A vs Z differences in gene expression reported here. The classification of genes was completed before microarray hybridization started, to eliminate bias in assignment of genes. Forty genes were classified as Z, 84 genes as A, and seven genes were equivocal and eliminated from further study. The linkage of 16 genes (10A, 6Z) based on Southern blot analysis was confirmed in metaphase chromosomes using fluorescent *in situ *hybridization of bacterial artificial chromosomes encoding the genes. Known 'ZW' genes – Z and W genes with highly similar sequences – were excluded from analysis.

The probe cDNAs were amplified by PCR to approximately 200 μg/μl and printed onto microslides using standard protocols. The Z, A, and several control genes were printed at least in duplicate and arrayed in random positions on the slides. Some genes were represented by more than one cDNA. Control genes such as glyceraldehyde-3-phosphate dehydrogenase (GAPDH) and β-actin were spotted 32–46 times, resulting in a small microarray with a random sample of A and Z genes, enriched in Z genes.

### Animals and RNA isolation

All procedures for animal use were approved by the University of California Los Angeles Chancellor's Animal Research Committee. To reduce the variability of the bird's environment, individual adult zebra finches were housed in separate cages within a large colony room for one week before collecting the tissues. During that period the finches could see and hear other finches. Birds were then moved to the laboratory and euthanized. Whole brain, liver, and kidney were dissected out and immediately frozen on dry ice. Whole brain was also collected in a similar manner from zebra finches within 24 hours of hatching. The sex of hatchlings was determined by PCR [12,47]. RNA was isolated using Trizol (Invitrogen, Carlsbad, CA) according to the manufacturer's instructions, and stored at -80°C.

### Zebra finch microarray hybridization and analyses

Total RNA from males and females was reverse transcribed. Samples of cDNA derived from one male and one female, one labeled with Cy3 and the other with Cy5, were mixed and dried in a vacuum centrifuge. Half of the male samples were labeled with Cy3, the others with Cy5. Each hybridization involved a competition of one male and one female sample, and each animal was analyzed in only one array. A total of nine male-female hybridizations were analyzed for adult brain, six for liver, nine for kidney, and eight for P1 brain. Hybridization was conducted using standard protocols. Slides were scanned using a G2505B Microarray Scanner (Agilent Technologies, Wilmington, DE). Array data are available from ArrayExpress [48] (accession numbers E-MEXP-543 and A-MEXP-308).

Hybridization signals were analyzed using ScanAlyze software [49]. Spots with weak or uneven signals were filtered out. To be included in the analysis, a spot had to escape such filtering in more than half of the male vs female hybridizations. The following numbers of genes were analyzed: adult brain 70 A and 29 Z, liver 56 A and 23 Z, kidney 73 A and 33 Z, and P1 brain 57 A and 25 Z.

For six genes we used quantitative RT-PCR to determine whether the sex differences detected with the microarray analysis were confirmed with another method (Table 3). Total RNA from the same individual males and female adult and P1 brains as used for microarray analysis (20 samples for adult brain and 16 samples for P1) was treated with RNase-free DNase I (Promega, Madison, WI) and first-strand cDNA was synthesized by oligo(dT) priming using Superscript III reverse transcriptase (Invitrogen). Gene-specific primers are shown in Additional data file 3. The exon-intron structure was checked in the chicken genome database [45] and the primers were designed to cross an intron, except for the follistatin gene, which had no intron inside the EST sequence. The amplified fragments were subcloned in pGEM-T Easy vector (Promega) and sequenced to confirm the identity of the product. Quantitative PCR was carried out with the SYBR Green PCR Master Mix (Applied Biosystems, Foster City, CA) and analyzed with an Applied Biosystems 7300 Real Time PCR System. In each case the dissociation curves of the amplified products confirmed the purity of the products. The expression of each gene was calculated from a standard curve and divided by the expression of GAPDH. GAPDH did not show sex differences in average expression in the four tissues (average M:F ratio in adult brain was 1.00, in kidney 0.994, in liver 0.999, and in P1 brain 0.991) in agreement with [9].

To compare the M:F ratio of expression of Z and A genes, the M:F expression ratio (ScanAlyze MRAT) for a spot was calculated as the median of the background-adjusted M:F ratios of hybridization for all pixels in the spot. The mean M:F ratio for all spots for each gene was calculated, and normalized to (divided by) the mean M:F ratio for the autosomal genes in the array. Mann-Whitney U tests were used to compare the M:F ratio of expression of Z vs autosomal genes in the four tissues.

To determine which genes showed a sex difference in expression, hybridization to each spot was background-corrected and normalized relative to the mean hybridization of all autosomal genes for that animal, averaged within an animal across all spots for each gene, and then compared between males and females with a paired *t*-test for each gene. Using the Q statistic [50], we set the false-discovery rate (FDR) at 10% in each tissue. To determine whether a gene was expressed at a significantly higher level in two or more tissues, we adopted a *t*-test value of *p *= 0.1 as the criterion for genes judged to be higher, because the probability that a gene reached that level in two tissues will typically be much smaller than 0.1 (for example, if expression in two tissues is independent, the probability would be (0.1)^2 ^= 0.01).

### Chicken array analyses

Twenty male and 20 female white leghorn embryos were harvested at 14 days of incubation. Brain, liver, and heart were removed, and RNA was extracted from each tissue using Trizol as described above. RNA samples were pooled (three to four animals per pool, five pools per sex). Hybridization to Affymetrix Chicken Arrays was performed by the UCLA DNA Microarray Core under the auspices of the NIH Neuroscience Microarray Consortium [51]. Array data are available from Gene Expression Omnibus (GEO) [52] (accession numbers GSE6843, GSE6844, GSE6856).

DChip software [53] was used for the normalization and filtration of the raw Affymetrix data. Following background subtraction, the invariant set normalization procedure was applied, which uses a subset of array probes with small differences between two arrays to fit the normalization curve. For each tissue, all arrays were normalized against the median male reference array because test normalizations showed no difference in the results when normalizing against male or female. To exclude genes that were not confidently detected in the arrays, we filtered out probes that had a less than 60% 'present' call in the arrays used. Normalized and filtered data were further analyzed using algorithms developed in R (version 2.10.2 [54]) with the help of R and Bioconductor packages including 'stats' (Fisher's Exact Test, KS test, Mann-Whitney U), 'genefilter' (*t*-test), 'qvalue' (FDR), and 'diptest' (Dip test). To reduce the number of genes represented twice in the probe list, we averaged expression values for probes that were identical in the first two parts of the probe ID (for example, the values for Gga.10049.1.A1_at and Gga.10049.1.S1_at were averaged). We excluded probes that had any missing hybridization values. Chromosomal linkage of probes was determined using 28 November 2006 Affymetrix annotations based on release 2.1 of the chicken genome [45]. Genes assigned to Unknown random, M, E22C19W28_E50C23, E22C19W28_E50C23_random, E64, E64_random, W and W random chromosomes were excluded from analysis. The final number of probe sets analyzed was 18,920 for brain, 16,506 for liver, and 17,757 for heart.

For seven chicken genes, we used quantitative RT-PCR to confirm the sex differences detected with the microarray analysis (Table 3 and Additional data file 3). Total RNA from the same sample for microarray (five samples for each sex, each sample contains four different animals) was tested using SYBR Green as described above for zebra finch. The expression of each gene was calculated from a standard curve and divided by the average value of expression for β_2_-microglobulin and β-actin. Sex differences in expression were analyzed with paired *t*-tests. The microarray and quantitative RT-PCR methods gave good agreement (see Table 3).

We analyzed the Z:A ratios of five microarray expression studies of other investigators utilizing 52 cDNA microarrays on chicken tissues. Raw values for A and Z genes were background-subtracted before calculating Z:A ratios. Array data were obtained from GEO database accessions GSE3347, GSE3723 [55], GSE3227, GSE3226 [56], and GSE1794. GSE3347 involved 12 arrays on 12 biological replicates from spleen and bursa of viral-loaded P24 chicks, containing 10,811 A genes and 405 Z genes. GSE3723 arrays on HTC avian macrophage cell lines stimulated with *Eimeria* contained 12 arrays with 11,265 A and 468 Z genes. GSE3227 arrays with four biological replicates, on embryonic day (E)10–17 pituitaries contained 16 arrays, with 4,446 A and 121 Z genes. GSE3226 arrays on E17-P3 pituitaries had 4,446 A genes and 121 Z genes and were made up of six arrays. GSE1794 arrays using macrophages derived from peripheral blood lymphocytes stimulated with whole *Escherichia coli *or lipopolysaccharide contained six arrays with 11,265 A genes and 468 Z genes.

### Mouse and human array analyses

The preparation of mouse tissues and the microarray analysis has been described [31]. Briefly, tissue was harvested from 169 female and 165 male adult mice that were an F2 cross of C3H and C57BL/6J strains. RNA was isolated from liver, gonadal adipose (epididymal fat pad in males, perimetrial fat pad in females), whole brain, and hamstring skeletal muscle RNA was reverse transcribed and labeled with Cy3 or Cy5. Individual female or male samples were hybridized against a pool of control cDNA. The microarrays contained 60mer oligonucleotides probes for 23,574 mouse genes and ESTs, and 2,186 control sequences (Agilent Technologies). Hybridization and transcript quantification were performed as previously described [57]. Individual transcript intensities were corrected for experimental variation and normalized, and were reported as the mean log_10 _ratio (mlratio) of an individual experiment relative to a pool from the F2 population [28,58]. A subset of the most actively expressed genes was selected for analysis [31] including 4,369 A and 134 X genes from brain, 12,299 A and 467 X from liver, 15,967 A and 610 X genes from adipose tissue, and 7,060 A and 277 X from muscle. The mouse microarray data are publicly available (GEO GPL2510, series GSE2814, GSE3086, GSE3087, GSE3088).

Gene-expression profiles of human lymphoblastoid cell lines from 15 CEPH/Utah families were obtained from GEO (record GDA1048, platform GPL564) based on [32]. In this dataset, the expression of 23,916 transcripts was measured using Agilent microarrays for 167 individuals (84 males and 83 females). Expression for each individual cell line was measured relative to a pool from all lines. Sex identifiers were obtained based on the pedigree information provided at [59]. Chromosomal linkage of transcripts was based on the GenBank accession IDs. A total of 12,041 A and 520 X genes were analyzed.

Gene-expression profiles of human hypothalamus were obtained from GEO (accession number GDS564) [34]. A total of five females and seven males were included in the study. Out of around 22,300 probes on the Affymetrix HG-U133A array, 13,625 transcripts were determined to be present in at least one female and one male (detection *p *< 0.01) and were subsequently used for our analysis. Among these selected transcripts, 469 were X and 11,508 were A.

The human muscle data were obtained from GEO GDS472 and GDS287 [35,36]. GDS472 contained data for 15 females and GDS287 listed data for 15 males. Affymetrix HG-U133A array was used for both sets. Among the 9,996 transcripts that were determined as being expressed (detection *p *< 0.01) in at least one female and one male, 369 were identified to be X and 9,427 were A.

The profiling data for human peripheral mononuclear cells were downloaded from the ArrayExpress database (array design accession number A-MEXP-170 and experiment accession number E-TABM-7) [33]. Ten females and eight males were included in the study. Because five data points were taken for each individual and there was strong consistency in gene expression across data points in the same individual [33], we averaged the intensity values of five data points for each transcript and selected 13,836 transcripts expressed (detection *p *< 0.01) in at least one female and one male. Among these, 517 were X and 11,618 were A.

## Additional data files

Additional data are available with this paper online. Additional data file 1 is a table of genes with significantly different expression in male vs female chick brain. Additional data file 2 is a table showing male:female ratios of expression of all genes in zebra finch. Additional data file 3 gives the primer sequences used for quantitative PCR.

## Supplementary Material

Additional data file 1 A table of genes with significantly different expression in male vs female chick brain Click here for file

Additional data file 2 A table showing male:female ratios of expression of all genes in zebra finch Click here for file

Additional data file 3 The primer sequences used for quantitative PCR Click here for file
