# Supplementary material for: Dosage compensation is less effective in birds than in mammals
Source: J Biol. 2007 Mar 22;6(1):2. doi: 10.1186/jbiol53 (PMC2373894; doi:10.1186/jbiol53)
Supplement: Additional data file 1 — A table of genes with significantly different expression in male vs female chick brain [file jbiol53-S1.pdf]

Table 4: Male to female ratios of expression of all genes in zebra finch
Genes differing between the sexes *p<0.05, **p<0.01, *** p<0.001.

Symbol	Genbank	Category	Adult brain		Kidney		Liver		P1 brain	
	DV958862	A	1.598		0.853		0.93		1.093	
MCCC2	CK310795	A	1.547	*	1.583	***	1.678			
PPIA	CK310626	A	1.515		0.957					
DAX1		A	1.458		1.083		1.271		1.321	
APBA1	DV955138	A	1.354	*	0.996				1.228	
SEPP1	CK306804	A	1.318		0.948		0.889		1.106	
CPLX2	CK314517	A	1.272		1.182		0.789		1.268	
TRPM7	DV961757	A	1.249	**	1.178		1.268		1.13	
UBAP2L	CK314627	A	1.234	***	1.166		1.156		1.325	*
RIOK2	CK310447	A	1.193		1.023		0.912		1.273	**
WNT4		A	1.192		0.98					
AR	AY847476	A	1.187		0.976		1.078			
DIAPH1	DV951868	A	1.181		0.977		1.014		0.939	
WDR51A	CK304055	A	1.172		0.923		1.04		1.189	*
	CK305660	A	1.168	***	1.066	*	0.94		1.154	***
PCSK5	DV955703	A	1.165		1.1		1.108			
SOX9		A	1.1		1.022					
PRKACB	DV959612	A	1.079		0.988		0.966			
LPL	CK313328	A	1.074		1.273					
RBM8B	CK307579	A	1.066		1.008		0.861		0.906	
cyclophillin		A	1.065	*	0.892		0.84		1.02	
COMMD10	CK311766	A	1.046		1.104	*	1.078		0.933	
ALDH1A2		A	1.043		1.164		0.89			
FRMD5	CK307621	A	1.039		0.924		0.64	*	0.893	
	CK303865	A	1.039		1.014		1.012		1.038	
SLC1A3	DV945569	A	1.016		1.055				1.067	
SMAD1	CK317055	A	1.005		0.825		1.202		1.132	
ST8SIA2	DV957129	A	1.003		0.902		1.077		0.862	
THBS4	DV951570	A	0.999		0.88		0.844		1.03	
GRIN1		A	0.991		0.878		0.935		1.139	
NFIC	CK305719	A	0.991		1.011		1.087		0.932	
HBLD2	CK309932	A	0.99		0.971		0.937		0.884	
ACOT12	DV951929	A	0.989		1.242					
RSBN1	DV955847	A	0.989		0.961		0.909		0.914	
	CK310834	A	0.988		1.143		0.997		0.992	
RORA	CK313162	A	0.988		1.02				0.969	
GPBP1L1	DV960486	A	0.986		1.155		1.204		0.888	
NOTCH1		A	0.976		1.014		0.888		1.147	*
KCNN2	CK235916	A	0.974		0.997		0.882		0.963	
ESR2		A	0.972		1.026		0.897		0.873	
	CK235138	A	0.966		1.098		0.928		0.806	

FBN1	CK304347	A	0.964		1.039					
NR5A1		A	0.961		0.932		0.976		1.065	*
ACTB		A	0.955		1.01		1.069		1.118	
	DV949309	A	0.952		1.046	*	0.906		0.881	
	CK312665	A	0.952		1.003		0.884		0.905	
GAPDH		A	0.949		0.922		1.011		0.898	
STOML2	CK303030	A	0.949		1.129		0.985		0.816	
TLE3	DV960301	A	0.942		1.124		1.205		0.955	
	DV956418	A	0.942		1.064	*	0.906		0.904	
	DV950172	A	0.942		1.407		1.018		1.065	
NRG2	CK303130	A	0.941		1.098		0.941		0.877	
MTX1	DV958545	A	0.941		0.968		0.96		0.913	
	CK301734	A	0.931		1.091		0.879		0.857	
	CK305333	A	0.931		1.071	*	0.96		0.936	
ARL1	CK303730	A	0.929		1.086		0.936		0.973	
TJP1	CK302684	A	0.926							
MAP1B	DV954903	A	0.924		1.127	*	0.981		0.924	
	CK312665	A	0.919		0.956		0.933		0.865	
SFRS11	CK305197	A	0.917		0.995		0.915		0.943	
	DV952782	A	0.914		0.983					
HSPB1	CK311542	A	0.913		1.001					
SOX3	DQ206644	A	0.912		0.949		0.963			
	CK312123	A	0.907		1.128		0.988		0.957	
RASA1	CK309014	A	0.901		1.038		0.882		0.798	*
	CK317038	A	0.9		0.975		0.865		1.058	
HDGF	CK301933	A	0.892		1.228				1.052	
CKMT1B	DV945274	A	0.874		0.935		1.064		0.829	
SH3GL3	CK311369	A	0.809	*	1.159				0.901	
VCP	DV951025	A	0.733	***	0.802		0.726	**	0.661	**
GNG11	CK305961	A							0.976	
DCX	DQ189989	A			0.926				1.105	
	DV946578	A			0.999					
BDNF		A			1.065					
XPA	DV954690	A			1.118					
GATA4		A								
GAS1	DV958631	A								
LHX9		A								
NANS	CK302389	A								
NGFB		A								
TGFB3		A								
WT1		A								
ESR1		A								
CYP19A1		A								
FST	CK304072	Z	1.831	***	1.139		1.492		1.658	*
RPS6	CK316148	Z	1.661	***	1.71	***	1.761	**	1.565	***
LUZP1	CK304237	Z	1.612	**	1.089				1.657	*

CBWD1	CK311497	Z	1.597	**	1.238				1.393	
HSD17B4	CK313884	Z	1.56	***	1.714	***	1.479	*	1.444	**
	CK313394	Z	1.559	**	1.625	***	1.615	*	1.582	**
	DV955869	Z	1.461	***	1.18	*	1.21			
PAIP1	DV959740	Z	1.409	*	1.011				0.943	
SMARCA2	DV958433	Z	1.4	***	1.363	**	1.514		1.366	
DNAJA1	CK315180	Z	1.374	***	1.333	**	1.545	***	1.121	**
TARS	DV946673	Z	1.358	*	1.146		1.32		1.44	
SLC30A5	CK306578	Z	1.337	***	1.079					
PSIP1	CK311614	Z	1.301		1.175				1.208	
	DV956828	Z	1.28	**	1.128		1.251		1.092	*
CRHBP	DV955207	Z	1.246		1.032					
TNPO1	DV948056	Z	1.239	*	1.18		1.019		1.055	*
ISL1	DV957275	Z	1.197		0.967		0.951		1.266	
	DV953757	Z	1.187	**	1.048		0.951		1.163	**
OXCT1	DV949695	Z	1.181	**	1.007				1.218	
MEF2C	CK316786	Z	1.168		0.786				1.008	
ERCC8	DV959526	Z	1.14	*	1.076		1.068		1.119	*
KIF27	DV959990	Z	1.129		0.92		0.984			
UHRF2	DV946458	Z	1.118	*	0.963		1.18		1.135	
C5orf18	CK315640	Z	1.056	**	0.99		0.995		0.972	
SMC2L1	DV954505	Z	1.042		1.091		1.002		1.432	*
NR2F1	CK306422	Z	1.018		1.118		0.93		0.916	
VLDLR	DV956173	Z	1.006		0.863		0.865		1.006	
HEXB	DV960732	Z	0.96		0.965		1.026		1.058	
AKAP2	CK309733	Z	0.948		0.944		0.947		0.909	
GHR		Z					1.07			
ACO1		Z			1.115		1.279			
DMRT1		Z			0.852					
DDX4	CK304812	Z			1.158					
	CK307964	Z			1.257					
CENPH	DV946684	Z								
ENC1	CK315833	Z								
MRPS27	CK304617	Z								
PAM	CK302180	Z								
NTRK2	AY679520	Z								
	CK303187	Z								
