# Supplementary material for: Dosage compensation is less effective in birds than in mammals
Source: J Biol. 2007 Mar 22;6(1):2. doi: 10.1186/jbiol53 (PMC2373894; doi:10.1186/jbiol53)
Supplement: Additional data file 3 — The primer sequences used for quantitative PCR [file jbiol53-S3.pdf]

**Table 6: Primer sequences for Quantitative PCR**

| | Chicken Affy # | Gene symbol | Primer sequences |
|---|---|---|---|
| Zebra finch | | FST | 5’-TGCCCGAGGAGTGTAATTCT-3’, 5’-GGTGCCACGTCCTACCTTTA-3’ |
| | | LUZP1 | 5’-CCTGTAGCTGCCAAGGAAGA-3’, 5’-AAAGCTTGGCATCTGATGGT-3’ |
| | | SMARCA2 | 5’-GGAGGATGGAGAAAAAGCAA-3’, 5’-CCGTCCTGAACTGTCCTTGT-3’ |
| | | DNAJA1 | 5’-ACCGGTGTAAGAGCTGCAAT-3’, 5’-ATATCCCCTGGTTCCAGACC-3’ |
| | | CRHBP | 5’-GGTGAAAATGACCCCTTCCT-3’, 5’-GTTTGCGGTGAAGGTGAACT-3’ |
| | | RPS6 | 5’-GAGAAAGCGCAAGTCTGTCC-3’, 5’-GTGTCAGTCAGCCCAGGAAT-3’ |
| | | GAPDH | 5’-CCATCAACGATCCCTTCATT-3’, 5’-TTCCCATTCTCAGCCTTGAC-3’ |
| Chicken | Gga.12454.1.S1 | | 5'-TTTGGCAGGAATGTGTTGCA-3', 5'-GTTTTCTTCCTCTGCACAGT-3' |
| | GgaAffx.9524 | MAP1B | 5'-TCATCCTACTATGTGGTGAG-3', 5'-CATTCCCTCATCACTTCTGA-3' |
| | GgaAffx.24493 | | 5'-TACTGTCGGTGTCCTTTTCA-3', 5'-TTGAGTACAGTCACTTGGCT-3' |
| | Gga.4811 | | 5'-CTAGTGAAGAACTGAAGGAG-3', 5'-ACATTACATCTGGTGGACAG-3' |
| | GgaAffx.25289 | RASA1 | 5'-TGGAAGGGGTAAATCCATTC-3', 5'-AGATCACGAGATAGGTCAGT-3' |
| | Gga.2433 | RELB | 5'-GCCTGTTGCAGAAACATTCA-3', 5'-CTGCTGGTTTTCAGATCAAG-3' |
| | Gga.2883 | DHFR | 5'-TAAAAGCCTGGATGATGCCT-3', 5'-CTTGTCACAAACAGTCGATG-3' |
| | | B2M | 5'-CTCCGACATGTCCTTCAACG-3', 5'-CTCGGGATCCCACTTGTAGA-3' |
| | | ACTB | 5'-TATTGCTGCGCTCGTTGTTG-3', 5'-GGGCGACCCACGATAGATG-3' |
